# Sex-Specific Associations Between Dynapenia and Risk of Atherosclerotic Cardiovascular Disease: A Machine-Learning-Based Approach

**DOI:** 10.3390/jpm15030083

**Published:** 2025-02-25

**Authors:** Gyumin Lee, Hye-Jin Kim, Heeji Choi, Seung-Ho Shin, Chulho Kim, Sang-Hwa Lee, Jong-Hee Sohn, Jae Jun Lee

**Affiliations:** 1 Artificial Intelligence Research Center, Hallym University Sacred Heart Hospital, Chuncheon 24253, Republic of Korea; weimdo_@naver.com (G.L.); khyejin1027@hanmail.net (H.-J.K.); choiheeji@hallym.or.kr (H.C.); sin12ho@gmail.com (S.-H.S.); 2 Department of Neurology, Hallym University College of Medicine, Chuncheon 24252, Republic of Korea; bleulsh@naver.com (S.-H.L.); deepfoci@hallym.or.kr (J.-H.S.); 3 Department of Anesthesiology, Hallym University College of Medicine, Chuncheon 24252, Republic of Korea; iloveu59@hallym.or.kr

**Keywords:** atherosclerotic cardiovascular disease, dynapenia, machine learning, sex difference, cardiovascular disease risk prediction

## Abstract

**Background/Objectives**: Dynapenia, age-associated loss in muscle strength, is an emerging risk factor for atherosclerotic cardiovascular disease (ASCVD), which may have different effects depending on sex. This study aims to investigate the association between dynapenia and ASCVD risk, evaluate its predictive significance among traditional factors, and explore sex-specific patterns through machine learning models. **Methods**: This retrospective case–control study uses data from 19,582 participants aged 40–79 from the Korean National Health and Nutrition Examination Survey (KNHANES). ASCVD risk is assessed using the American College of Cardiology/American Heart Association 10-year risk algorithm, with dynapenia defined based on hand grip strength. Multivariable logistic regression and ML algorithms, including light gradient boosting (LGB) and XGBoost (XGB), are applied to examine predictive factors. Model performance is evaluated via the area under the receiver operating characteristic curve (AUROC), and Shapley additive explanation (SHAP) analysis highlights variable importance. **Results**: Dynapenia prevalence is higher in women (33.4%) than men (13.9%) at high ASCVD risk. Logistic regression shows dynapenia is significantly associated with high ASCVD risk in women (odds ratio, 1.47; 95% confidence interval, 1.20–1.81) but not in men. Machine learning models demonstrate excellent predictive performance, with XGB achieving the highest AUROC (0.950 in men and 0.963 in women). The SHAP analysis identifies dynapenia as a critical risk factor in women, while body mass index, educational status, and household income are influential in both sexes. **Conclusions**: Dynapenia is a significant ASCVD risk factor in women, emphasizing sex-specific prevention strategies. Machine learning enhances risk assessment precision, underscoring muscle health’s role in cardiovascular care.

## 1. Introduction

Atherosclerotic cardiovascular disease (ASCVD) is a major global health burden, so increasing attention is being paid to non-traditional risk factors such as physical health and inflammation [1]. Sarcopenia, characterized by the loss of muscle mass, is a known risk factor for ASCVD [2]. The decline in muscle strength with age—known as dynapenia—has emerged as an even more robust predictor of ASCVD risk [3]. Dynapenia, characterized by reduced muscle strength, influences ASCVD risk through mechanisms such as systemic inflammation or decreased physical activity [4,5].

Dynapenia has been linked to oxidative stress, mitochondrial dysfunction, and skeletal muscle motor unit alterations due to physical inactivity. However, its relationship with cardiovascular disease remains poorly understood [6,7]. Several studies have reported associations between dynapenia and adverse cardiovascular outcomes. For example, in a cohort of 7030 individuals from the English Longitudinal Study of Ageing, Ramírez et al. demonstrated that individuals with both dynapenia and abdominal obesity had a 1.85-fold increased risk of cardiovascular mortality compared to those without [8]. Additionally, Uchida et al. observed a higher prevalence of dynapenia in women (43.3%) than in men (29.2%), highlighting sex disparities in its impact on cardiovascular health [9].

In addition to advancing age, sex is an important factor affecting the body distribution of skeletal muscle mass [10]. Therefore, when investigating the association of ASCVD with dynapenia, various patient factors such as smoking, marital status, and physical activity, which influence the development of dynapenia, should be considered simultaneously [11]. Sex is an important factor to stratify when assessing the association of dynapenia with cardiovascular outcomes, but most studies have either included sex in the model as an adjusting variable or investigating the interactions with other variables [12,13]. In addition, a variety of variables, including lifestyle risk behaviors, have been reported to be associated with ASCVD outcomes. However, these variables are also strongly influenced by age and sex, meaning that when investigating the association between dynapenia and ASCVD outcomes, models should be selected that allow for multiple interactions between variables. Machine learning (ML) provides advanced tools to identify complex relationships between emerging predictors like dynapenia and traditional risk factors. In addition, these ML methods have the advantage of not having to consider the interaction between various variables [14]. Therefore, in this study, we aimed to analyze the effect of dynapenia on the development of ASCVD by sex and to analyze whether various factors, including dynapenia, are different in predicting ASCVD risk by sex using ML methods. Our study aimed to determine whether dynapenia is an independent predictor of ASCVD risk, leveraging ML techniques to improve predictive accuracy and to identify potential sex-specific differences in its impact.

## 2. Materials and Methods

### 2.1. Study Design

This is a retrospective case–control study using nationwide data from the Korean National Health and Nutrition Examination Survey (KNHANES) [15]. The KNHANES data include health examinations such as handgrip strength measurements, nutritional surveys, and anthropometric measurements. Participants were recruited using stratified, multistage, and probability sampling methods to reflect the characteristics of a representative population in Korea, and all patients participated in the survey after informed consent was obtained. All data are stored in a publicly accessible repository and can be downloaded after obtaining institutional approval from the KNHANES database administrators.

### 2.2. Participants

**Figure 1:**
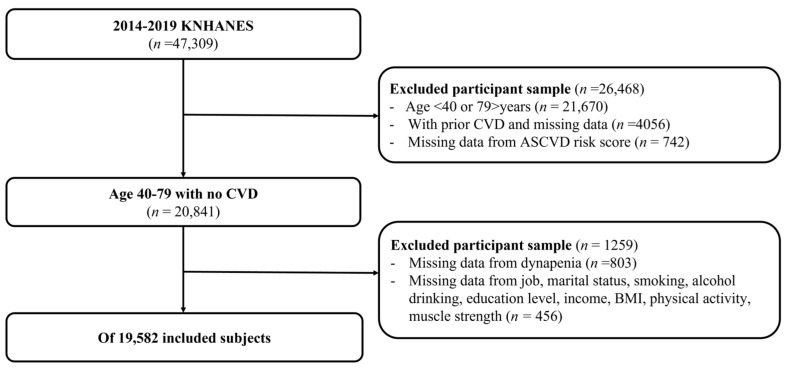
Flow chart of study participants. KNHANES, Korean National Health and Nutrition Examination Survey; CVD, cardiovascular disease; ASCVD, atherosclerotic cardiovascular disease.

Among the 47,309 participants who participated in the KNHANES survey from 2014 to 2019, we included those aged 40 to 79 years for whom an ASCVD score could be calculated. We excluded individuals with a history of CVD, those lacking records on CVD status, and those without sufficient data for ASCVD score calculation. From the 20,841 patients without a history of CVD, we further excluded those lacking muscle strength measurements or data on variables related to dynapenia, such as physical activity or nutritional status (Figure 1). The study protocol and data collection procedures were approved by the Institutional Review Board of the KNHANES or affiliated research institutions (IRB No.: 2024-10-010).

### 2.3. Definition of Variables

Patients’ occupations were categorized into manual workers, non-manual workers, and others, including those without an occupation. Manual or non-manual workers were classified based on standard job classification criteria [16]. Household income was divided into quartiles, and educational year was categorized into 4 groups: less than 6 years, 6–9 years, 9–12 years, and more than 12 years. Marital status was categorized as having a spouse or not, with the latter including widowed, divorced, and never married individuals. Regions were categorized as urban based on administrative districts or rural for those living in ‘eup’ or ‘myeon’, which correspond to city-level and town-level areas, respectively. Alcohol drinking was defined as drinking alcohol more than twice per week, and smoking was defined as having smoked more than 100 cigarettes in one’s lifetime. Physical activity was categorized into three levels (low, moderate, high) based on the International Physical Activity Questionnaire, scored in metabolic equivalent task minutes as the scoring metric. Cancer and depression were defined as conditions diagnosed by a physician.

### 2.4. Muscle Strength Measurement

Muscle strength was assessed using hand grip strength, measured with a digital hand dynamometer (T.K.K 5401, Takei Scientific Instruments Co., Ltd., Tokyo, Japan). Muscle strength was measured twice for each hand, alternating between the right and left. The highest recorded value was used to represent the participant’s strength. Dynapenia was defined according to the 2019 Asian Working Group for Sarcopenia criteria: hand grip strength < 28 kg for men and <18 kg for women [17].

### 2.5. Assessment of Atherosclerotic Cardiovascular Disease Risk

The 10-year ASCVD risk was assessed based on the criteria established by the American College of Cardiology/American Heart Association [18]. Although the ASCVD risk score estimation was originally developed for white populations, it has been applied to other ethnic groups as well [19]. Additionally, the 10-year ASCVD risk is categorized as <5% (lowest), 5–10% (low), 10–15% (moderate), and >15% (high) [20]. However, we dichotomized ASCVD at 15% because numerous studies suggest that ASCVD scores derived from Western populations tend to underestimate risk in Asians. For analysis, the ASCVD risk score was dichotomized using a threshold of 15% [21,22].

### 2.6. Statistical Analysis

Descriptive statistics for participant characteristics by sex are presented as mean ± standard deviation for continuous variables and as proportion (percent) for categorical variables. Comparisons between groups based on ASCVD risk in both sexes were performed using Mann–Whitney U test for continuous variables and χ^2^ test for categorical variables. To analyze the association between dynapenia and ASCVD risk, we first performed a binary logistic regression analysis with ASCVD risk as the outcome variable. Variables with *p* < 0.05 in the univariable analysis were included as covariates in the multivariable model. Statistical significance was assessed using odds ratios (ORs) and 95% confidence intervals (CIs).

Second, high ASCVD risk was classified using logistic regression, support vector machine, random forest, extreme gradient boost (XGB), and light gradient boosting (LGB) models. The dataset was split into training and test datasets in an 8:2 ratio, and the proportion of dynapenia was equally allocated in the training and test datasets. The input features of the ML model included all the variables used in the logistic regression analysis. Among these, the variables that overlapped with the ASCVD risk score were age, sex, low-density lipoprotein cholesterol, and current smoking. Nominal variables were entered directly into the ML models, while continuous variables were normalized through min–max scaling before training. A 5-fold cross-validation was conducted to evaluate the generalization performance of the models. We conducted nested cross-validation on the training dataset and reserved the test dataset only for evaluating the final model. There was no missing data in the ML model preprocessing and no additional outlier correction. The LGB model is a tree-based model and had a learning rate of 0.05, 1000 trees, and a maximum depth of 5. The XGB model is also a tree-based model and had a learning rate of 0.05, 1000 trees, and a maximum depth of 5. The SVM model used the RBF kernel with a C-value of 1.0. The random forest model was set to 1000 trees with a maximum depth of 5. For the loss function, we used binary cross-entropy. The Optuna optimization algorithm was employed to identify the best hyperparameters (Table S1). The area under the receiver operating characteristic curve (AUROC) was used to assess the models’ discriminative ability, with results stratified by sex. Training parameters were optimized for AUROC. To interpret the ML model results, the Shapley value-based explainable artificial intelligence approach was used to visualize variable importance. Additionally, accuracy, precision, recall, F1 score, specificity, and area under the precision–recall curve were calculated to comprehensively evaluate model performance. The source code for all ML models can be found in the Supplemental Data (Text S1).

## 3. Results

### 3.1. Descriptive Statistics

This dataset included data from 19,582 participants. The baseline characteristics of the groups after the final exclusion of patients did not differ significantly from those of the entire cohort, including the excluded patients (Table S2). The mean age was 57.4 ± 10.7 years, and 43% of the participants were men. Figure S1 illustrates the correlation between age and ASCVD risk score in men and women, showing a positive association that remained consistent across the sexes, particularly at younger ages. Figure S2 presents the correlation between muscle strength and ASCVD risk score by sex, revealing a negative association in both men and women. Notably, ASCVD risk increased as muscle strength decreased, especially within the dynapenia-defined range. High ASCVD risk was more prevalent in men than in women (33.0% vs. 13.1%, Table 1). ASCVD risk was higher among both men and women who were non-manual workers compared to manual workers or others, had lower household incomes, had lower educational levels, lived in rural areas rather than urban areas, or had been diagnosed with cancer or depression. Marital status was not associated with ASCVD risk in men but was linked to higher ASCVD risk in women who did not have a spouse. Physical activity, specifically engaging in strength training two or more days per week, was associated with lower ASCVD risk in women compared to men. The prevalence of dynapenia was significantly higher in women (33.4%) than in men (13.9%) with high ASCVD risk. The presence of dynapenia was also associated with higher ASCVD risk in both men and women.

### 3.2. Assessment of High ASCVD Risk

Table 2 presents the results of a binary logistic regression analysis of the variables, including dynapenia, for high ASCVD risk stratified by sex. The multivariable analysis revealed that household income and years of education were negatively associated with high ASCVD risk, while BMI was positively associated. When the variables used in the ASCVD risk calculation were excluded, job status, marital status, and residential area (rural vs. urban) were associated with high ASCVD risk, with sex-specific differences. In men, being in the “other” job category was associated with higher ASCVD risk compared to manual workers. In women, marital status was a significant factor, with those without a spouse having a higher ASCVD risk, whereas higher physical activity levels were associated with a lower ASCVD risk. In the multivariable analysis, dynapenia showed a nonsignificant association with high ASCVD risk in men. However, in women, dynapenia was significantly and positively associated with high ASCVD risk (OR, 1.46; 95% CI, 1.19–1.80).

### 3.3. ML Performance for High ASCVD Risk

**Figure 2:**
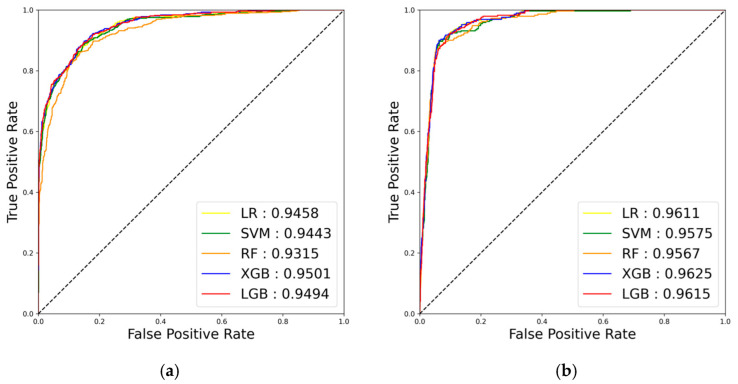
Model performance in predicting high atherosclerotic cardiovascular disease risk in men (**a**) and women (**b**) presented as area under the receiver operating characteristic curve. LR, logistic regression; SVM, support vector machine; RF, random forest; XGB, extreme gradient boosting; LGB, light gradient boosting.

Figure 2 illustrates the AUROC performance results by sex on the test dataset across the five ML algorithms trained to predict high ASCVD risk. Among both men and women, the XGB model demonstrated the best performance (AUROC, 0.950; 95% CI, 0.940–0.960 for men; AUROC, 0.963; 95% CI, 0.954–0.971 for women) in predicting high ASCVD risk. Detailed performance metrics stratified by sex are presented in Table 3. The XGB model achieved the highest AUROC and performed well across other metrics. Specifically, both precision and recall were high, indicating strong predictive performance for both positive and negative cases. Additionally, as shown in Table 1, the XGB model maintained a high area under the precision–recall curve score even with increased class imbalance, particularly due to the lower proportion of women at high ASCVD risk. This further supported the selection of the XGB model, as it outperformed other ML models despite the class imbalance.

**Figure 3:**
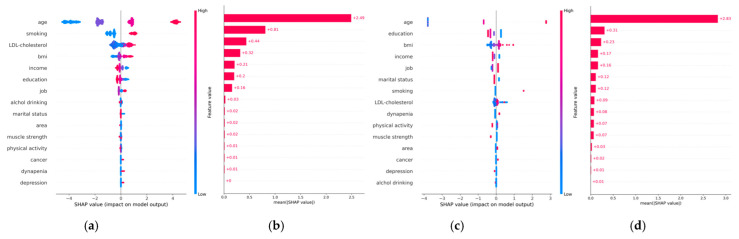
Shapley additive explanation (SHAP) values in ML models for high atherosclerotic cardiovascular disease risk prediction according to sex. Feature importance plot in men (**a**) and in women (**c**). SHAP summary plot of extreme gradient boosting model in men (**b**) and women (**d**).

Figure 3 shows the SHAP values for the best-performing XGB models by sex. In men, the most important predictors of high ASCVD risk were age, current smoking, low-density lipoprotein cholesterol, BMI, household income, years of education, job status, and alcohol consumption, in that order. In women, dynapenia was identified as an important variable associated with ASCVD, after age, years of education, body mass index, household income, marital status, current smoking, and low-density lipoprotein cholesterol.

## 4. Discussion

This study aimed to investigate the relationship between dynapenia and ASCVD risk, with a specific focus on sex-based differences. Our findings provide novel insights into the contribution of dynapenia to increased ASCVD risk, particularly in women. Using a large, nationally representative sample from the KNHANES database, this study highlights the critical role of muscle strength in cardiovascular health, emphasizing sex differences. Additionally, the sex-based differences in dynapenia were further supported by the explanatory power of the variables in the ML analysis. Logistic regression analysis assumes a linear relationship between variables, but ML models can capture non-linear relationships. In real-world datasets, there can be complex non-linear relationships between variables, so we were able to further confirm the association between dynapenia and ASCVD risk through ML models that can account for these relationships. In addition, it can be difficult to design the effect of interactions directly in logistic regression models, whereas ML models can automatically learn the interactions between these variables. Additionally, ML methodologies can be advantageous for learning complex patterns among multiple variables and improving predictive performance. In addition, although the odds ratio from logistic regression and the SHAP value from our ML models are not directly comparable, examining both provides a different perspective on which variables, including dynapenia, are associated with ASCVD risk.

In this study, we analyzed the association between dynapenia and cardiovascular disease using ASCVD risk estimates rather than actual cardiovascular events. ASCVD risk estimates indicate the likelihood of a cardiovascular event occurring but do not directly predict cardiovascular events. Additionally, this study examined whether there was a sex-specific association between dynapenia and ASCVD risk, rather than assessing whether dynapenia was a significant predictor of ASCVD. Despite these limitations, ASCVD risk estimates can serve as a useful tool for the initial assessment of cardiovascular disease risk and prevention. The observed association of dynapenia with ASCVD risk estimates suggests that dynapenia may also influence the occurrence of actual cardiovascular events. Therefore, the relationship between dynapenia and cardiovascular events should be further validated in future prospective cohort studies using actual cardiovascular events as outcome variables.

The findings from our descriptive analysis indicate that high ASCVD risk is significantly more prevalent in men than in women. This aligns with the existing literature suggesting that traditional risk factors, such as age, smoking, and hypertension, contribute more substantially to ASCVD in men [23]. However, when assessing the impact of dynapenia, we observed a much higher prevalence of low muscle strength in women with high ASCVD risk compared to men. These findings are consistent with previous studies reporting a higher incidence of dynapenia in women, particularly with aging [24]. The higher prevalence of dynapenia in women may reflect sex-specific differences in muscle mass, hormonal factors, or lifestyle patterns. These differences warrant further investigation to better understand the underlying mechanisms. In addition, we found that women with a spouse had a higher ASCVD risk compared to those without a spouse. Some studies suggest that women without a spouse tend to prioritize their health more, engaging in regular physical activity and maintaining a healthy diet [25]. These lifestyle factors may contribute to a lower risk of cardiovascular disease. However, this may be a specific trend, as ASCVD risk can be influenced by various factors, including individual lifestyle, social support, and economic status.

Our logistic regression analysis revealed noteworthy findings regarding the relationship between dynapenia and ASCVD risk. While dynapenia was not significantly associated with ASCVD risk in men, it showed a strong positive association in women. Specifically, women with dynapenia had 46% higher odds of being at high ASCVD risk compared to those without dynapenia. This significant finding underscores the potentially unique role of dynapenia in women’s cardiovascular health. The mechanisms underlying this relationship are likely multifactorial. Reduced muscle strength may reflect a broader decline in physical activity, a well-established contributor to cardiovascular risk [26]. Furthermore, dynapenia may exacerbate systemic inflammation, a key driver of atherosclerosis [27]. Hormonal differences, particularly the effects of estrogen on muscle mass and cardiovascular health, may also contribute to women’s higher susceptibility to dynapenia-associated ASCVD risk [28].

The lack of a significant association between dynapenia and ASCVD risk in men may be attributed to several factors. First, men generally have a higher baseline muscle mass than women, making their muscle strength more resilient to declines associated with aging or other factors [21]. Additionally, traditional risk factors such as smoking and hypertension may exert a stronger influence on ASCVD risk in men, potentially overshadowing the effects of dynapenia [9]. Another consideration is the threshold used to define dynapenia (i.e., hand grip strength < 28 kg for men), which may not fully capture the nuances of muscle weakness in men, particularly in older populations.

In this study, to exclude younger participants, individuals under 40 years of age were not included. Furthermore, factors that may influence strength loss, such as body mass index, smoking, alcohol consumption, and physical activity, were included as covariates in the logistic regression and ML model. The KNHANES study already excluded subjects with severe malnutrition or nutritional disorders that could interfere with physical measurements, as well as those with severe activity limitations, such as being bedridden or having muscle damage, during the recruitment phase. Furthermore, factors that may influence strength loss, such as body mass index, smoking, alcohol consumption, and physical activity, were included as covariates in the logistic regression and ML model.

Our ML analysis further supported the findings from logistic regression. The models demonstrated good predictive performance with the XGB model for both men and women in predicting high ASCVD risk. SHAP analysis, which evaluates variable importance, revealed that traditional risk factors such as BMI, household income, and education were significant predictors in both sexes. However, dynapenia emerged as a particularly important predictor of high ASCVD risk in women. This suggests that while lifestyle factors remain crucial for both men and women, dynapenia may serve as an important indicator of increased ASCVD risk in women, potentially reflecting other underlying risk factors. Therefore, incorporating dynapenia into risk assessments alongside other factors could enhance early detection and prevention strategies, particularly for women. However, it is important to recognize that dynapenia may act as a surrogate marker for other underlying risk factors rather than a direct cause of increased ASCVD risk.

The XGB model offers several advantages over models like LGB, random forest, and support vector machine. The XGB model often achieves state-of-the-art results in various ML challenges due to its robust handling of complex data patterns and interactions [29]. Its built-in regularization techniques help prevent overfitting, enhancing model generalization. While the LGB model is known for its faster training speed and efficiency, the XGB model tends to provide superior predictive accuracy, making it a preferred choice when performance is critical [30]. Compared to random forest, which builds multiple independent decision trees, the XGB model constructs trees sequentially, each aiming to correct the errors of its predecessors. This sequential boosting approach allows XGB to outperform random forest by effectively capturing complex relationships within the data. In any case, the high performance of all five ML models in predicting high ASCVD risk on our test dataset is significant evidence that dynapenia is associated with ASCVD risk.

In our results, we used nested internal validation with 5-fold cross validation. In other words, after randomly dividing the training dataset into five different folds, four folds were used for training, and the remaining one fold was used as the validation set. In addition, the test dataset was 20% of the total dataset, which was not used for training but only for the evaluation of the final model. Nevertheless, this study did not perform an external test comparison, so the results of this study do not fully guarantee the generalization performance of the model. Further studies with external validation using other nationwide prospective cohort data are needed to support these findings.

Due to the limitations of the dataset, this study used only grip strength measurements to diagnose dynapenia using the AWGS 2019 criteria. The grip strength measurements were not further standardized or normalized. It is important to consider that grip strength alone may not accurately assess all aspects of dynapenia and that strength and the physical performance of other muscle groups may be more important, especially in older adults. Therefore, caution should be used in interpreting the results of this study, and future studies should consider other measures of strength and physical performance in addition to grip strength.

This study has several strengths. First, the use of a large, nationally representative dataset enhances the generalizability of the findings to the broader population. The inclusion of multiple ML algorithms enabled the evaluation of predictive accuracy across different models, with cross-validation providing a robust assessment of model performance. Second, the focus on sex-based differences offers valuable insights into how sex-specific factors influence the relationship between muscle strength and cardiovascular disease. Lastly, we demonstrated that the sex-specific association between ASCVD risk and dynapenia observed in conventional logistic regression analysis was equally significant in ML analysis, as confirmed by the SHAP values.

However, this study has several limitations. As a retrospective observational study, it cannot establish causality between dynapenia and ASCVD risk. The cross-sectional nature of the data also limits the ability to determine the temporal sequence of events. While we controlled for many potential confounders, unmeasured factors such as dietary habits, medications, and genetic predispositions may still influence both muscle strength and cardiovascular health. Future longitudinal studies are needed to confirm the causal relationship between dynapenia and ASCVD risk and to investigate underlying mechanisms. Additionally, assessing muscle strength through hand grip measurement, although widely used, may not fully reflect overall muscle function, particularly in older adults or individuals with comorbid conditions. Lastly, this study analyzed ASCVD risk as a surrogate marker rather than actual cardiovascular events. While this may be a limitation, it further highlights the association of dynapenia with factors that influence ASCVD risk.

In summary, this study highlights sex differences in the association between dynapenia and cardiovascular disease risk. For women, the prevention and improvement of dynapenia should focus on regular strength training for at least 30 min, three or more times per week, along with a protein-rich diet. For men, actively managing traditional risk factors—such as smoking, high blood pressure, and high cholesterol—while maintaining a healthy lifestyle is crucial. These sex-specific prevention strategies can contribute to more effective cardiovascular disease prevention and management.

## 5. Conclusions

This study suggests a significant relationship between dynapenia and ASCVD risk, particularly in women. The significant association between dynapenia and high ASCVD risk in women underscores the need for further research into sex-specific interventions to improve muscle strength and prevent cardiovascular disease. ML models showed promise in assessing dynapenia and sex-specific ASCVD risk. As we continue to explore the complex relationships between muscle health and cardiovascular outcomes, dynapenia should be considered a key factor in the prevention and management of ASCVD, especially in older women.

## Figures and Tables

**Table 1:** Sex-specific descriptive characteristics of participants according to the atherosclerotic cardiovascular disease risk (10-year risk < 15% vs. ≥15%).

	Men (*n* = 8442)			Women (*n* = 11,140)		
	ASCVD < 15% (*n* = 5659)	ASCVD ≥ 15% (*n* = 2783)	*p* Value	ASCVD < 15% (*n* = 9684)	ASCVD ≥ 15% (*n* = 1456)	*p* Value
Age, years			<0.001			<0.001
40–59	4494 (79.4)	317 (11.4)		6564 (67.8)	6 (0.4)	
60–69	1115 (19.7)	1087 (39.1)		2619 (27.0)	180 (12.4)	
70–79	50 (0.9)	1379 (49.6)		501 (5.2)	1270 (87.2)	
Job status			<0.001			<0.001
manual workers	2031 (35.9)	236 (9.5)		1790 (18.5)	15 (1.0)	
non-manual workers	2931 (51.8)	1214 (43.6)		3710 (38.3)	381 (26.2)	
other workers	697 (12.3)	1306 (46.9)		4184 (43.2)	1060 (72.8)	
Household income			<0.001			<0.001
low	467 (8.3)	909 (32.7)		1470 (15.2)	838 (57.6)	
low-middle	1242 (21.9)	870 (31.3)		2456 (25.4)	330 (22.7)	
high-middle	1726 (30.5)	560 (20.1)		26.82 (27.7)	179 (12.3)	
high	2224 (39.3)	444 (16.0)		3076 (31.8)	109 (7.5)	
Educational status			<0.001			<0.001
≤elementary school	485 (8.6)	978 (35.1)		2084 (21.5)	1117 (76.7)	
middle school	616 (10.9)	511 (18.4)		1329 (13.7)	163 (11.2)	
high school	1959 (34.6)	788 (28.3)		3533 (36.5)	130 (8.9)	
≥college	2599 (45.9)	506 (18.2)		2738 (28.3)	46 (3.2)	
Marital status			0.376			<0.001
with spouse	4941 (87.3)	2410 (86.6)		1851 (19.1)	761 (52.3)	
without spouse	718 (12.7)	373 (13.4)		7833 (80.9)	695 (47.7)	
Residential area			<0.001			<0.001
rural	4593 (81.2)	2063 (74.1)		8011 (82.7)	1021 (70.1)	
urban	1066 (18.8)	720 (25.9)		1673 (17.3)	435 (29.9)	
Alcohol drinking			0.763			<0.001
<2/week	3436 (60.7)	1700 (61.1)		8666 (89.5)	1377 (94.6)	
≥2/week	2223 (39.3)	1083 (38.9)		1018 (10.5)	79 (5.4)	
Current smoking			0.666			0.851
no	3756 (66.4)	1861 (66.9)		9291 (95.9)	1399 (96.1)	
yes	1903 (33.6)	922 (33.1)		393 (4.1)	57 (3.9)	
Physical activity			0.530			<0.001
<600	2550 (45.1)	1287 (46.2)		4660 (48.1)	917 (63.0)	
600 ≤ MET-week/min < 1500	1598 (28.2)	758 (27.2)		2988 (30.9)	365 (25.1)	
≥1500	1511 (26.7)	738 (26.5)		2036 (21.0)	174 (12.0)	
Muscle strength exercise			1.000			<0.001
<2 day	4052 (71.6)	1992 (71.6)		8211 (84.8)	1341 (92.1)	
≥2 day	1607 (28.4)	791 (28.4)		1473 (15.2)	115 (7.9)	
Cancer			<0.001			<0.001
no	5454 (96.4)	2530 (90.9)		9014 (93.1)	1311 (90.0)	
yes	205 (3.6)	253 (9.1)		670 (6.9)	145 (10.0)	
Depression			<0.001			0.009
no	5546 (98.0)	2700 (97.0)		9078 (93.7)	1338 (91.9)	
yes	113 (2.0)	83 (3.0)		606 (6.3)	118 (8.1)	
LDL, mg/dL	117.4 (96.9–138.6)	110.3 (87.0–133.0)	<0.001	120.2 (98.8–143.0)	109.8 (85.0–135.4)	<0.001
BMI, kg/m^2^ *	24.3 (22.5–26.3)	24.2 (22.3–26.1)	0.031	23.3 (21.4–25.7)	24.5 (22.6–26.7)	<0.001
Dynapenia *			<0.001			<0.001
no	5512 (97.4)	2395 (86.1)		8771 (90.6)	969 (66.6)	
yes	147 (2.6)	388 (13.9)		913 (9.4)	487 (33.4)	

* Median (interquartile range). ASCVD, atherosclerotic cardiovascular disease; MET, metabolic equivalent task; LDL, low-density lipoprotein; BMI, body mass index.

**Table 2:** Association between dynapenia and atherosclerotic cardiovascular disease risk in binary logistic regression analysis.

	OR (95% CI)	
	Men	Women
Age, years		
40–59	1.00 (reference)	1.00 (reference)
60–69	20.55 (17.0–24.84)	53.91(23.32–124.62)
70–79	674.66 (472.30–963.74)	1609.2 (692.7–3438.3)
Job status		
manual workers	1.00 (reference)	1.00 (reference)
non-manual workers	1.01 (0.81–1.25)	0.60 (0.30–1.22)
other workers	1.56 (1.24–1.98)	0.90 (0.45–1.81)
Household income		
low	1.00 (reference)	1.00 (reference)
low-middle	0.71 (0.57–0.59)	0.79 (0.63–0.98)
high-middle	0.58 (0.46–0.74)	0.72 (0.55–0.94)
high	0.53 (0.42–0.68)	0.72 (0.52–1.00)
Educational status		
≤elementary school	1.00 (reference)	1.00 (reference)
middle school	0.85 (0.67–1.07)	0.73 (0.56–0.95)
high school	0.72 (0.59–0.89)	0.70 (0.53–0.92)
≥college	0.50 (0.39–0.64)	0.50 (0.33–0.78)
Marital status		
with spouse	1.00 (reference)	1.00 (reference)
without spouse	1.03 (0.82–1.28)	0.67 (0.56–0.80)
Residential area		
rural	1.00 (reference)	1.00 (reference)
urban	0.95 (0.80–1.13)	1.25 (1.02–1.55)
Alcohol drinking		
<2/week	1.00 (reference)	1.00 (reference)
≥2/week	1.08 (0.93–1.25)	1.04 (0.71–1.53)
Current smoking		
no	1.00 (reference)	1.00 (reference)
yes	4.77 (4.01–5.67)	5.11 (3.17–8.22)
Physical activity		
<600	1.00 (reference)	1.00 (reference)
600 ≤ MET-week/min < 1500	0.91 (0.77–1.09)	0.95 (0.77–1.17)
≥1500	0.88 (0.73–1.04)	0.72 (0.56–1.05)
Muscle strength exercise		
<2 day	1.00 (reference)	1.00 (reference)
≥2 day	1.03 (0.87–1.21)	0.75 (0.56–1.02)
Cancer		
no	1.00 (reference)	1.00 (reference)
yes	1.11 (0.82–1.49)	1.11 (0.82–1.50)
Depression		
no	1.00 (reference)	1.00 (reference)
yes	1.05 (0.66–1.64)	0.76 (0.56–1.05)
LDL, mg/dl	1.00 (1.00–1.01)	1.00 (0.99–1.00)
BMI, kg/m^2^	1.16 (1.13–1.19)	1.07 (1.04–1.10)
Dynapenia		
no	1.00 (reference)	1.00 (reference)
yes	1.10 (0.801–1.521)	1.46 (1.19–1.80)

OR, odds ratio; CI, confidence interval; ASCVD, atherosclerotic cardiovascular disease; MET, metabolic equivalent task; LDL, low-density lipoprotein; BMI, body mass index.

**Table 3:** Predictive model performance for atherosclerotic cardiovascular disease risk by sex on the test dataset.

Model	LR	SVM	RF	XGB	LGB
Men					
Accuracy	0.863 (0.846–0.879)	0.865 (0.849–0.881)	0.858 (0.841–0.875)	0.886 (0.871–0.900)	0.869 (0.853–0.885)
Precision	0.757 (0.729–0.785)	0.750 (0.724–0.778)	0.746 (0.718–0.774)	0.879 (0.852–0.906)	0.763 (0.736–0.790)
Recall	0.860 (0.831–0.889)	0.885 (0.858–0.910)	0.864 (0.835–0.892)	0.758 (0.722–0.794)	0.876 (0.849–0.903)
F1 score	0.805 (0.782–0.827)	0.812 (0.791–0.833)	0.800 (0.778–0.822)	0.814 (0.788–0.839)	0.815 (0.794–0.837)
AUROC	0.946 (0.935–0.956)	0.944 (0.933–0.955)	0.932 (0.918–0.944)	0.950 (0.940–0.960)	0.949 (0.939–0.959)
Specificity	0.864 (0.844–0.883)	0.855 (0.834–0.875)	0.855 (0.835–0.875)	0.949 (0.936–0.961)	0.866 (0.846–0.885)
AUPRC	0.912 (0.894–0.928)	0.911 (0.893–0.927)	0.888 (0.869–0.907)	0.918 (0.902–0.933)	0.918 (0.902–0.933)
Women					
Accuracy	0.922 (0.911–0.933)	0.930 (0.920–0.941)	0.924 (0.913–0.935)	0.935 (0.956–0.945)	0.930 (0.912–0.940)
Precision	0.645 (0.609–0.683)	0.677 (0.641–0.716)	0.656 (0.620–0.696)	0.714 (0.676–0.755)	0.679 (0.641–0.719)
Recall	0.904 (0.869–0.935)	0.893 (0.856–0.928)	0.880 (0.842–0.918)	0.842 (0.797–0.883)	0.873 (0.835–0.911)
F1 score	0.753 (0.723–0.783)	0.770 (0.740–0.800)	0.752 (0.721–0.784)	0.773 (0.740–0.806)	0.764 (0.732–0.795)
AUROC	0.961 (0.951–0.970)	0.958 (0.947–0.967)	0.957 (0.947–0.966)	0.963 (0.954–0.971)	0.961 (0.953–0.970)
Specificity	0.925 (0.913–0.937)	0.936 (0.925–0.946)	0.931 (0.920–0.942)	0.949 (0.940–0.959)	0.938 (0.927–0.948)
AUPRC	0.763 (0.717–0.811)	0.753 (0.706–0.801)	0.744 (0.694–0.767)	0.770 (0.723–0.816)	0.750 (0.700–0.803)

## Data Availability

All data are stored in a publicly accessible repository and can be downloaded after obtaining institutional approval from the KNHANES database administrators.

## Supplementary Materials

The following supporting information can be downloaded at: https://www.mdpi.com/article/10.3390/jpm15030083/s1, Table S1: Extreme gradient boosting tuning parameters of the best model predicting high ASCVD risk in men and women; Table S2: Comparison of baseline characteristics between the final study population and participants with missing variables; Figure S1: Result of scatter plot association between age and atherosclerotic cardiovascular disease risk score in men (A) and women (B); Figure S2: Results of correlation between muscle strength and atherosclerotic cardiovascular disease risk score in men (A) and women (B); Text S1: Python code for this study.

## Abbreviations

The following abbreviations are used in this manuscript:

ASCVD	Atherosclerotic cardiovascular disease
CVD	Cardiovascular disease
ML	Machine learning
KNHANES	Korean National Health and Nutrition Examination Survey
XGB	Extreme gradient boosting
LGB	Light gradient boosting
AUROC	Area under the receiver operating characteristic curve
SHAP	Shapley additive explanation

## References

[1] Lechner K., von Schacky C., McKenzie A.L., Worm N., Nixdorff U., Lechner B., Kränkel N., Halle M., Krauss R.M., Scherr J. (2020). Lifestyle factors and high-risk atherosclerosis: Pathways and mechanisms beyond traditional risk factors. Eur. J. Prev. Cardiol..

[2] Damluji A.A., Alfaraidhy M., AlHajri N., Rohant N.N., Kumar M., Al Malouf C., Bahrainy S., Ji Kwak M., Batchelor W.B., Forman D.E. (2023). Sarcopenia and cardiovascular diseases. Circulation.

[3] Kim D., Lee J., Park R., Oh C.M., Moon S. (2024). Association of low muscle mass and obesity with increased all-cause and cardiovascular disease mortality in US adults. J. Cachexia Sarcopenia Muscle.

[4] de Luca Corrêa H., dos Santos Rosa T., Dutra M.T., Sales M.M., Noll M., Deus L.A., Reis A.L., de Araújo T.B., Neves R.V.P., Gadelha A.B. (2021). Association between dynapenic abdominal obesity and inflammatory profile in diabetic older community-dwelling patients with end-stage renal disease. Exp. Gerontol..

[5] Sampaio R.A.C., Sampaio P.Y.S., Uchida M.C., Arai H. (2020). Management of dynapenia, sarcopenia, and frailty: The role of physical exercise. J. Aging Res..

[6] Chainy G.B., Sahoo D.K. (2020). Hormones and oxidative stress: An overview. Free Radic. Res..

[7] Ribeiro J.C., Duarte J.G., Gomes G.A., Costa-Guarisco L.P., de Jesus I.T., Nascimento C.M., Santos-Orlandi A.A., Orlandi F.S., Vasilceac F.A., Zazzetta M.S. (2021). Associations between inflammatory markers and muscle strength in older adults according to the presence or absence of obesity. Exp. Gerontol..

[8] Ramírez P.C., de Oliveira D.C., de Oliveira Máximo R., de Souza A.F., Luiz M.M., Delinocente M.L.B., Steptoe A., de Oliveira C., da Silva Alexandre T. (2023). Is dynapenic abdominal obesity a risk factor for cardiovascular mortality? A competing risk analysis. Age Ageing.

[9] Uchida S., Kamiya K., Hamazaki N., Nozaki K., Ichikawa T., Nakamura T., Yamashita M., Maekawa E., Reed J.L., Yamaoka-Tojo M. (2021). Prognostic utility of dynapenia in patients with cardiovascular disease. Clin. Nutr..

[10] Janssen I., Heymsfield S.B., Wang Z., Ross R. (2000). Skeletal muscle mass and distribution in 468 men and women aged 18–88 yr. J. Appl. Physiol..

[11] Lee M., Ahn H.-J., Lee S.J., Kim P.-J., Kim C., Lee S.-H., Sohn J.-H., Lee J.-J. (2024). Lifestyle risk behavior and atherosclerotic cardiovascular risk: An analysis using the Korea National Health and Nutrition Examination Survey. PLoS ONE.

[12] Setoyama Y., Honda T., Tajimi T., Sakata S., Oishi E., Furuta Y., Shibata M., Hata J., Kitazono T., Nakashima Y. (2024). Association between dynapenic obesity and risk of cardiovascular disease: The Hisayama study. J. Cachexia Sarcopenia Muscle.

[13] Rahimi Farahani M., Sharifi F., Payab M., Shadman Z., Fakhrzadeh H., Moodi M., Khorashadizadeh M., Ebrahimpur M., Taheri M., Ebrahimi P. (2024). Dynapenia-abdominal obesity and mortality risk, is independent effect obscured by age and frailty?: Birjand Longitudinal Aging Study (BLAS). J. Diabetes Metab. Disord..

[14] Jabeur S.B., Mefteh-Wali S., Viviani J.-L. (2024). Forecasting gold price with the XGBoost algorithm and SHAP interaction values. Ann. Oper. Res..

[15] Kweon S., Kim Y., Jang M.-j., Kim Y., Kim K., Choi S., Chun C., Khang Y.-H., Oh K. (2014). Data resource profile: The Korea national health and nutrition examination survey (KNHANES). Int. J. Epidemiol..

[16] Son M., Ye B.J., Kim J.-I., Kang S., Jung K.-Y. (2015). Association between shift work and obesity according to body fat percentage in Korean wage workers: Data from the fourth and the fifth Korea National Health and Nutrition Examination Survey (KNHANES 2008–2011). Ann. Occup. Environ. Med..

[17] Chen L.-K., Woo J., Assantachai P., Auyeung T.-W., Chou M.-Y., Iijima K., Jang H.C., Kang L., Kim M., Kim S. (2020). Asian Working Group for Sarcopenia: 2019 consensus update on sarcopenia diagnosis and treatment. J. Am. Med. Dir. Assoc..

[18] Goff D.C., Lloyd-Jones D.M., Bennett G., Coady S., D’agostino R.B., Gibbons R., Greenland P., Lackland D.T., Levy D., O’donnell C.J. (2014). 2013 ACC/AHA guideline on the assessment of cardiovascular risk: A report of the American College of Cardiology/American Heart Association Task Force on Practice Guidelines. Circulation.

[19] Chia Y.C., Lim H.M., Ching S.M. (2014). Validation of the pooled cohort risk score in an Asian population–a retrospective cohort study. BMC Cardiovasc. Disord..

[20] Henderson K., Kaufman B., Rotter J.S., Stearns S., Sueta C.A.A., Foraker R., Michael Ho P., Chang P.P. (2022). Socioeconomic status and modification of atherosclerotic cardiovascular disease risk prediction: Epidemiological analysis using data from the atherosclerosis risk in communities study. BMJ Open.

[21] Selvarajah S., Kaur G., Haniff J., Cheong K.C., Hiong T.G., van der Graaf Y., Bots M.L. (2014). Comparison of the Framingham Risk Score, SCORE and WHO/ISH cardiovascular risk prediction models in an Asian population. Int. J. Cardiol..

[22] Rana J.S., Tabada G.H., Solomon M.D., Lo J.C., Jaffe M.G., Sung S.H., Ballantyne C.M., Go A.S. (2016). Accuracy of the atherosclerotic cardiovascular risk equation in a large contemporary, multiethnic population. J. Am. Coll. Cardiol..

[23] Bays H.E., Taub P.R., Epstein E., Michos E.D., Ferraro R.A., Bailey A.L., Kelli H.M., Ferdinand K.C., Echols M.R., Weintraub H. (2021). Ten things to know about ten cardiovascular disease risk factors. Am. J. Prev. Cardiol..

[24] Mitchell W.K., Williams J., Atherton P., Larvin M., Lund J., Narici M. (2012). Sarcopenia, dynapenia, and the impact of advancing age on human skeletal muscle size and strength; a quantitative review. Front. Physiol..

[25] Li W., Xu Z., Tang W. (2024). Gender differences in self-rated health among older adults in the Chinese workforce. Front. Public Health.

[26] Aubertin-Leheudre M., Anton S., Beavers D.P., Manini T.M., Fielding R., Newman A., Church T., Kritchevsky S.B., Conroy D., McDermott M.M. (2017). Dynapenia and metabolic health in obese and nonobese adults aged 70 years and older: The LIFE study. J. Am. Med. Dir. Assoc..

[27] Pan L., Xie W., Fu X., Lu W., Jin H., Lai J., Zhang A., Yu Y., Li Y., Xiao W. (2021). Inflammation and sarcopenia: A focus on circulating inflammatory cytokines. Exp. Gerontol..

[28] García-Alfaro P., García S., Rodríguez I., Pérez-López F.R. (2022). Handgrip strength, dynapenia, and related factors in postmenopausal women. Menopause.

[29] Zhang H., Eziz A., Xiao J., Tao S., Wang S., Tang Z., Zhu J., Fang J. (2019). High-resolution vegetation mapping using eXtreme gradient boosting based on extensive features. Remote Sens..

[30] Zhang D., Gong Y. (2020). The comparison of LightGBM and XGBoost coupling factor analysis and prediagnosis of acute liver failure. IEEE Access.

