# Supplementary material for: Sex-Specific Associations Between Dynapenia and Risk of Atherosclerotic Cardiovascular Disease: A Machine-Learning-Based Approach
Source: J Pers Med. 2025 Feb 25;15(3):83. doi: 10.3390/jpm15030083 (PMC11942907; doi:10.3390/jpm15030083)
Supplement: Supplementary file 1 [file jpm-15-00083-s001.zip › jpm-3446382-supplementary.pdf]

Table S1 1. Extreme gradient boosting tuning parameters of the best predicting model of high ASCVD risk in men and women.

Parameters	Search space	Selected value	
		Men	Women
Booster	gbtree	gbtree	gbtree
Class_weight	balanced	balanced	balanced
Max_depth	3,4,5,6,7,8,9,10	9	9
Colsample_bytree	0.5,0.6,0.7,0.8,0.9,1.0	1.0	1.0
Gamma	0,1	0	0
Learning_rate	0.1,0.01,0.05	0.05	0.05
Min_child_weight	1, 2, 3	1	1
N_estimators	10, 3000	87	87

Table S2. Comparison of baseline characteristics between the final study population and participants with missing variables.

		Including missing data		Final population data		P-value
Sex	men	9078	(42.1%)	8442	(43.1%)	1.000
	women	12505	(57.9%)	11140	(56.9%)	
Age, year	40-59	12282	(56.9%)	11381	(58.1%)	0.199
	60-69	5499	(25.5%)	5001	(25.5%)	
	70-79	3802	(17.6%)	3200	(16.3%)	
Job status	manual workers	4308	(20.1%)	4099	(20.9%)	0.199
	non manual workers	8976	(41.9%)	8236	(42.1%)	
	other workers	8155	(38.0%)	7247	(37.0%)	
Household income	low	4322	(20.1%)	3684	(18.8%)	0.213
	low-middle	5424	(25.2%)	4898	(25.0%)	
	high-middle	5552	(25.8%)	5147	(26.3%)	
	high-middle	6212	(28.9%)	5853	(29.9%)	
Educational status	≤elementary school	5424	(25.3%)	4664	(23.8%)	0.213
	middle school	2903	(13.5%)	2619	(13.4%)	
	high school	6892	(32.1%)	6410	(32.7%)	
	≥college	6240	(29.1%)	5889	(30.1%)	
Marital status	with spouse	4239	(19.7%)	3703	(18.9%)	1.000
	without spouse	17335	(80.4%)	15879	(81.1%)	
Residential area	Rural	17205	(79.7%)	15688	(80.1%)	1.000
	Urban	4378	(20.9%)	3894	(19.9%)	
Alcohol drinking	<2/week	16702	(77.9%)	15179	(77.5%)	1.000
	≥2/week	4737	(22.1%)	4403	(22.5%)	
Current smoking	No	17868	(83.4%)	16307	(83.3%)	1.000
	Yes	3551	(16.6%)	3275	(16.7%)	
Physical activity (MET-week/min)	<600	10403	(48.6%)	9414	(48.1%)	0.199
	600-1500	6186	(28.9%)	5709	(29.2%)	
	≥1500	4837	(22.6%)	4459	(22.8%)	
Muscle strength exercise	<2day	17255	(80.3%)	15596	(79.6%)	1.000
	≥2day	4229	(19.7%)	3986	(20.4%)	
Cancer	no	20122	(93.2%)	18309	(93.5%)	1.000
	yes	1458	(6.8%)	1273	(6.5%)	
Depression	no	20474	(94.9%)	18662	(95.3%)	1.000
	yes	1105	(5.1%)	920	(4.7%)	
Dynapenia	no	18469	(89.6%)	17647	(90.1%)	1.000

	yes	2145	(10.4%)	1935	(9.9%)	
High ASCVD risk	no	16127	(78.1%)	15343	(78.4%)	1.000
	yes	4526	(21.9%)	4239	(21.6%)	
BMI, kg/m^2		23.85	(21.85-26.04)	23.85	(21.87-26.03)	0.833
LDL, mg/dl		117	(95.58-140)	117	(96-140)	0.639

MET, metabolic equivalent of task; ASCVD, atherosclerotic cardiovascular disease; BMI, body mass index; LDL, low-density lipoprotein cholesterol.

Figure S1. Result of scatter plot association between age and atherosclerotic cardiovascular disease risk score in male (A) and female (B).

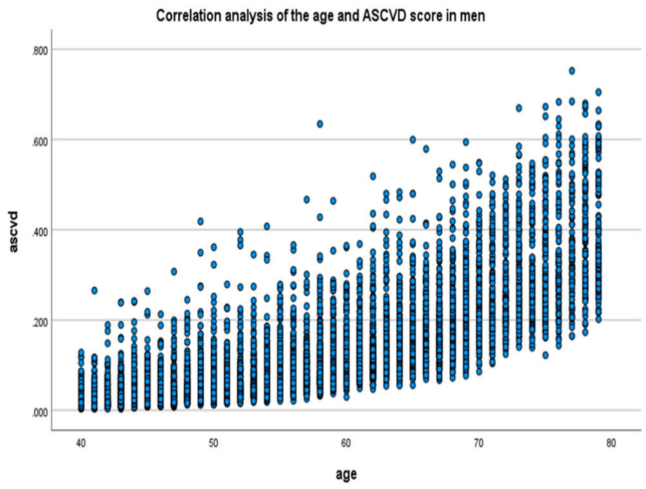

(a)

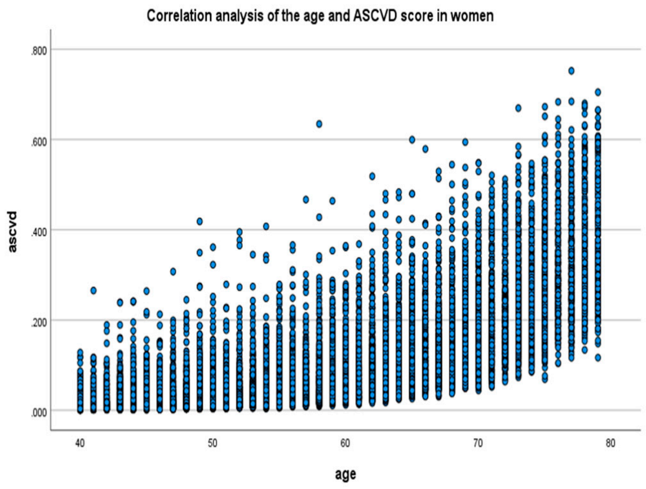

(b)

ascvd: atherosclerotic cardiovascular disease

Figure S2. Result of correlation between muscle strength and atherosclerotic cardiovascular disease risk score in male (A) and female (B).

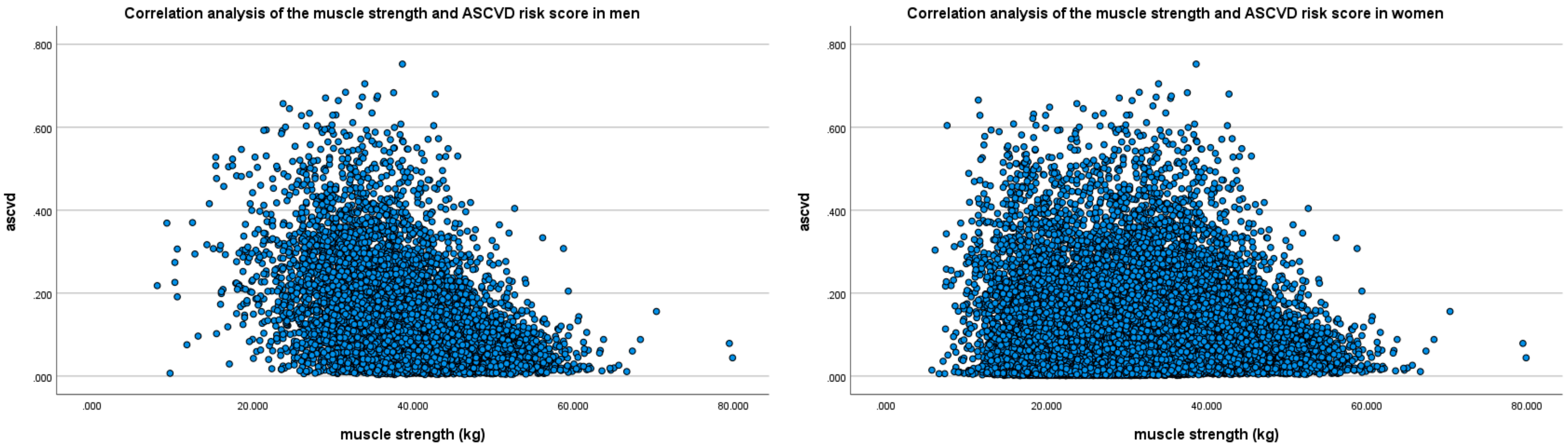

ascvd: atherosclerotic cardiovascular disease

Text T1. Python codes for our manuscript.

```
import os
os.environ['TF_CPP_MIN_LOG_LEVEL']='3'
import warnings
warnings.filterwarnings('ignore')

import random
import numpy as np
import pandas as pd
import seaborn as sns
import matplotlib.pyplot as plt
import scipy
import imblearn

from sklearn.metrics import *
from sklearn.metrics import precision_recall_curve, auc
from sklearn.pipeline import Pipeline, make_pipeline
from sklearn.linear_model import LogisticRegression
from sklearn.svm import SVC
from sklearn.base import BaseEstimator, TransformerMixin, RegressorMixin, clone
from sklearn.model_selection import train_test_split, StratifiedKFold,
GridSearchCV
from sklearn.linear_model import LogisticRegression
from sklearn.svm import SVC
from sklearn.feature_selection import RFE
from sklearn.metrics import average_precision_score
from sklearn import preprocessing
from sklearn.ensemble import RandomForestClassifier
from sklearn.feature_selection import SelectKBest, f_classif
from sklearn.metrics import roc_auc_score
from sklearn.metrics import precision_recall_curve

#XGBOOST
from xgboost import XGBClassifier
from lightgbm import LGBMClassifier

import optuna
from optuna import Trial

def normalize(df):
    result = df.copy()
    for feature_name in df.columns:
        max_value = df[feature_name].max()
        min_value = df[feature_name].min()
         result[feature_name] = (df[feature_name] - min_value) / (max_value -
min_value)
    return result
```

```
def custom_scaler(df,method):
    if method == 'minmax':
        df = normalize(df)
        print('Scaler : minmax')
    else:
        print('Uncertain Method')
    return df

def custom_target(X,y, target, valid_size):
    if y.columns[0] == target[0]:
        X = X
        y = y
        print('target : ', target)
    else:
        print('Uncertain Method')

        X_train, X_test, Y_train, Y_test = train_test_split(X, y,
test_size=valid_size, stratify=y)
    return X_train, X_test, Y_train, Y_test

def class_ratio(y_train, y_test):
    class_ratio=y_train.value_counts()
    print("Train Dataset Class Ratio")
    for i in range(len(class_ratio)):
        print("Class ",i," : ",class_ratio[i])

    print("Test Dataset Class Ratio")
    test_class_ratio=y_test.value_counts()
    for i in range(len(test_class_ratio)):
        print("Class ",i," : ",test_class_ratio[i])









def get_clf_eval(Y_test, pred, pred_proba, model ,df ,folder_path):
    # confusion = confusion_matrix(Y_test, pred)
    ACC = accuracy_score(Y_test, pred)
    Precision = precision_score(Y_test, pred)
    Recall = recall_score(Y_test, pred)
    F1_score = f1_score(Y_test, pred)
    Specificity = imblearn.metrics.specificity_score(Y_test, pred)
    ROC_AUC = roc_auc_score(Y_test, pred_proba[:])
```

```
    AUPRC = average_precision_score(Y_test, pred_proba[:])


    print('ACC: {0:.4f}, Precision: {1:.4f}, Recall: {2:.4f}, F1 score: {3:.4f}, 
ROC_AUC: {4:.4f}, Specificity: {5:.4f}, AUPRC: {6:.4f}'.format(
        ACC, Precision, Recall, F1_score, ROC_AUC, Specificity, AUPRC))

     perfomance  =  pd.DataFrame(data=[[str(model)[:str(model).find('(')],  ACC, 
Precision, Recall, F1_score, ROC_AUC, Specificity, AUPRC]],
                              columns=['Model' ,'Accuracy' ,'Precision' ,'Reca
ll' ,'F1_score' , 'ROC_AUC', 'Specificity', 'AUPRC'])
    total = pd.concat([df,perfomance],axis=0)

    return total

def objective_lr(trial :Trial, X_train ,Y_train ,X_test ,Y_test):

    strtfdKFold = StratifiedKFold(n_splits=5)
    kfold = strtfdKFold.split(X_train, Y_train)

    param = {
        'penalty' : trial.suggest_categorical('penalty', ['l2']),
        'C' : trial.suggest_float('C', 0.01, 10, step=0.1) ,
        'max_iter' : trial.suggest_int('max_iter', 1, 5000),
    }

    LR = LogisticRegression(**param,
                            class_weight='balanced'
                            )

    for k, (train, test) in enumerate(kfold):
        LR.fit(X_train.iloc[train, :], Y_train.iloc[train])

    pred = LR.predict_proba(X_test)
    pred = pred.astype(np.float64)
    log_score = log_loss(Y_test, pred ,normalize=True)

    return log_score

def best_study_LR(X_train, X_test, Y_train, Y_test, LR_parms, df, folder_path):

    strtfdKFold = StratifiedKFold(n_splits=5)
    kfold = strtfdKFold.split(X_train, Y_train)

    lr = LogisticRegression(penalty=LR_parms['penalty'],
                            C=LR_parms['C'],
                            max_iter=LR_parms['max_iter'],
                            class_weight='balanced',
```

```python
                              verbose=0
                              #class_weight=class_weight
                              )
    for k, (train, test) in enumerate(kfold):

        lr.fit(X_train.iloc[train, :], Y_train.iloc[train])
    lr_pred_test = lr.predict(X_test)
    lr_pred_test_prob = lr.predict_proba(X_test)[:, 1]

    lr_result = get_clf_eval(Y_test, lr_pred_test, lr_pred_test_prob, lr, df,
folder_path)
    get_feature_importance(X_train, lr, folder_path)
    get_confusion_matrix(Y_test, lr_pred_test, "LR", folder_path)

    return lr,lr_pred_test_prob, lr_result

def objective_svm(trial:Trial,X_train,Y_train,X_test,Y_test):

    strtfdKFold = StratifiedKFold(n_splits=5)
    kfold = strtfdKFold.split(X_train, Y_train)

    param = {
        'kernel' : trial.suggest_categorical('kernel', ['linear']),
        'C' : trial.suggest_float('C', 0.01, 10, step=0.01),
    }
    svc = SVC(**param,
              class_weight='balanced',
              probability=True)
    for k, (train, test) in enumerate(kfold):
        svc.fit(X_train.iloc[train, :], Y_train.iloc[train])

    pred = svc.predict(X_test)
    pred = pred.astype(np.float64)
    log_score = log_loss(Y_test, pred,normalize=True)

    return log_score

def   best_study_SVM(X_train,   X_test,   Y_train,   Y_test,   SVC_parms,   df,
folder_path):
    strtfdKFold = StratifiedKFold(n_splits=5)
    kfold = strtfdKFold.split(X_train, Y_train)

    svc = SVC(kernel=SVC_parms['kernel'],
              C=SVC_parms['C'],
              #gamma=SVC_parms['gamma'],
              class_weight='balanced',
              #class_weight=class_weight,
              probability=True,
```

```
            verbose=0)

    for k, (train, test) in enumerate(kfold):
        svc.fit(X_train.iloc[train, :], Y_train.iloc[train])

    svc_pred_test = svc.predict(X_test)
    svc_pred_test_prob = svc.predict_proba(X_test)[:, 1]

     svc_result = get_clf_eval(Y_test, svc_pred_test, svc_pred_test_prob, svc,
df, folder_path)
    get_feature_importance(X_train, svc, folder_path)
    get_confusion_matrix(Y_test, svc_pred_test, "SVC", folder_path)

    return svc,svc_pred_test_prob, svc_result

def objective_rf(trial: Trial, X_train, Y_train, X_test, Y_test):
    param = {
         'max_depth': trial.suggest_int('max_depth',2,20),
         'max_leaf_nodes': trial.suggest_int('max_leaf_nodes',2,20),
         'min_samples_leaf': trial.suggest_int('min_samples_leaf',1,200),
         'n_estimators': trial.suggest_int('n_estimators',10,1000),
    }

    strtfdKFold = StratifiedKFold(n_splits=5)
    kfold = strtfdKFold.split(X_train, Y_train)

    rf = RandomForestClassifier(**param,
                        class_weight='balanced',
                        #class_weight=class_weight,
                        verbose=0)

    for k, (train, test) in enumerate(kfold):
        rf.fit(X_train.iloc[train, :], Y_train.iloc[train])

    rf_preds = rf.predict_proba(X_test)
    rf_preds = rf_preds.astype(np.float64)
    log_score = log_loss(Y_test, rf_preds, normalize=True)

    return log_score

def best_study_RF(X_train, X_test, Y_train, Y_test, rf_parms, df, folder_path):
    result_auc = []

    strtfdKFold = StratifiedKFold(n_splits=5)
    kfold = strtfdKFold.split(X_train, Y_train)

    for k, (train, test) in enumerate(kfold):
        rf = RandomForestClassifier(max_depth=rf_parms['max_depth'],
```

```
                            max_leaf_nodes=rf_parms['max_leaf_nodes'],
                            min_samples_leaf = rf_parms['min_samples_leaf'],
                            n_estimators = rf_parms['n_estimators'],
                            class_weight='balanced',
                            verbose=0
                            )
        rf.fit(X_train.iloc[train, :], Y_train.iloc[train])
        rf_pred = rf.predict(X_train.iloc[test, :])
        rf_CM = confusion_matrix(rf_pred, Y_train.iloc[test])
        rf_AUC = roc_auc_score(Y_train.iloc[test], rf_pred)
        result_auc.append(rf_AUC)

    rf_pred_test = rf.predict(X_test)
    rf_pred_test_prob = rf.predict_proba(X_test)[:, 1]

     result  =  get_clf_eval(Y_test,  rf_pred_test,  rf_pred_test_prob,  rf,  df,
folder_path)
    get_feature_importance(X_train, rf, folder_path)
    get_confusion_matrix(Y_test, rf_pred_test, "RF", folder_path)

    return rf,rf_pred_test_prob, result

def   objective_xgb(trial:   Trial,   X_train,   Y_train,   X_test,   Y_test,
enable_categorical=True):
    param = {
        'booster' : trial.suggest_categorical('booster', ['gbtree']),
        'max_depth': trial.suggest_int('max_depth',1,10),
        'subsample': trial.suggest_float("subsample", 0.05, 1.0),
        'colsample_bytree': trial.suggest_float("colsample_bytree", 0.05, 1.0),
            'scale_pos_weight':  trial.suggest_categorical('scale_pos_weight',
[1,2,3,4]),
        'lambda': trial.suggest_float("lambda", 1e-8, 2.0, log=True),
        'alpha': trial.suggest_float("alpha", 1e-8, 2.0, log=True),
               'learning_rate':   trial.suggest_float("learning_rate",   0.01,
0.05),
        'n_estimators': trial.suggest_int('n_estimators',100,2000),
        'gamma':trial.suggest_int('gamma', 0, 1),
        'min_child_weight': trial.suggest_int("min_child_weight", 1, 20),
        'max_bin': trial.suggest_int("max_bin", 2, 512),
    }

    strtfdKFold = StratifiedKFold(n_splits=5)
    kfold = strtfdKFold.split(X_train, Y_train)

    xgb = XGBClassifier(**param,
                        class_weight='balanced',
                        # class_weight=class_weight,
                        verbosity=0)
```

```
    for k, (train, test) in enumerate(kfold):
                    xgb.fit(X_train.iloc[train,    :],    Y_train.iloc[train],
eval_set=[(X_train.iloc[test, :],Y_train.iloc[test])], verbose=0)
    xgb_preds = xgb.predict_proba(X_test)
    xgb_preds = xgb_preds.astype(np.float64)
    log_score = log_loss(Y_test, xgb_preds, normalize=True)

    return log_score

def   best_study_XGB(X_train,   X_test,   Y_train,   Y_test,   XGB_parms,   df,
folder_path):
    result_auc = []

    strtfdKFold = StratifiedKFold(n_splits=5)
    kfold = strtfdKFold.split(X_train, Y_train)

    for k, (train, test) in enumerate(kfold):
        xgb = XGBClassifier(booster=XGB_parms['booster'],
                            max_depth=XGB_parms['max_depth'],
                            subsample=XGB_parms['subsample'],
                            colsample_bytree = XGB_parms['colsample_bytree'],
                            scale_pos_weight = XGB_parms['scale_pos_weight'],
                            reg_alpha = XGB_parms['alpha'],
                            reg_lambda = XGB_parms['lambda'],
                            gamma = XGB_parms['gamma'],
                            n_estimators=XGB_parms['n_estimators'],
                            learning_rate=XGB_parms['learning_rate'],
                            min_child_weight=XGB_parms['min_child_weight'],
                            max_bin=XGB_parms['max_bin'],
                            class_weight='balanced',
                            verbosity=0
                            )
             xgb.fit(X_train.iloc[train,  :],  Y_train.iloc[train],  eval_set=
[(X_train.iloc[test, :],Y_train.iloc[test])], verbose=0)
        xgb_pred = xgb.predict(X_train.iloc[test, :])
        xgb_CM = confusion_matrix(xgb_pred, Y_train.iloc[test])
        xgb_AUC = roc_auc_score(Y_train.iloc[test], xgb_pred)
        result_auc.append(xgb_AUC)

    xgb_pred_test = xgb.predict(X_test)
    xgb_pred_test_prob = xgb.predict_proba(X_test)[:, 1]

     result = get_clf_eval(Y_test, xgb_pred_test, xgb_pred_test_prob, xgb, df,
folder_path)
    get_feature_importance(X_train, xgb, folder_path)
    get_confusion_matrix(Y_test, xgb_pred_test, "XGB", folder_path)
```

```
    return xgb,xgb_pred_test_prob, result

def objective_lgb(trial: Trial, X_train, Y_train, X_test, Y_test):
    param = {
                'boosting_type'   :   trial.suggest_categorical('boosting_type',
['gbdt','dart','rf','goss']),
        "n_estimators" : trial.suggest_int('n_estimators',100,2000),
        'max_depth':trial.suggest_int('max_depth', 1, 10),
        'reg_alpha': trial.suggest_loguniform('reg_alpha', 1e-8, 1.0),
        'reg_lambda': trial.suggest_loguniform('reg_lambda', 1e-8, 1.0),
        'num_leaves': trial.suggest_int('num_leaves', 31, 200),
           'colsample_bytree':  trial.suggest_uniform('colsample_bytree',  0.1,
1.0),
        'subsample': trial.suggest_uniform('subsample', 0.5, 1.0),
        'min_data_in_leaf': trial.suggest_int('min_data_in_leaf', 20, 200),
        'min_child_samples': trial.suggest_int('min_child_samples', 1, 3),
        'learning_rate': trial.suggest_float("learning_rate", 0.01, 0.05),
         'early_stopping_round': trial.suggest_int('early_stopping_round', 50,
50)
    }

    strtfdKFold = StratifiedKFold(n_splits=5)
    kfold = strtfdKFold.split(X_train, Y_train)

    lgb = LGBMClassifier(**param,
                        class_weight='balanced',
                        verbosity=0
                        )

    for k, (train, test) in enumerate(kfold):
                                        lgb.fit(X_train.iloc[train,        :],
Y_train.iloc[train],  eval_metric='logloss',eval_set=[(X_train.iloc[test, :],Y
_train.iloc[test])]) #

    lgb_preds = lgb.predict_proba(X_test)
    lgb_preds = lgb_preds.astype(np.float64)
    log_score = log_loss(Y_test, lgb_preds, normalize=True)

    return log_score

def   best_study_lgb(X_train,   X_test,   Y_train,   Y_test,   lgb_parms,   df,
folder_path):
    result_auc = []

    strtfdKFold = StratifiedKFold(n_splits=5)
    kfold = strtfdKFold.split(X_train, Y_train)

    for k, (train, test) in enumerate(kfold):
```

```
        lgb = LGBMClassifier(boosting_type=lgb_parms['boosting_type'],
                             max_depth=lgb_parms['max_depth'],
                             subsample=lgb_parms['subsample'],
                             colsample_bytree = lgb_parms['colsample_bytree'],
                             min_data_in_leaf = lgb_parms['min_data_in_leaf'],
                             reg_alpha = lgb_parms['reg_alpha'],
                             reg_lambda = lgb_parms['reg_lambda'],#
                             num_leaves = lgb_parms['num_leaves'],
                             n_estimators=lgb_parms['n_estimators'],
                             learning_rate=lgb_parms['learning_rate'],
                             min_child_samples=lgb_parms['min_child_samples'],
                             class_weight='balanced',
                                                       early_stopping_round   =
lgb_parms['early_stopping_round'],
                             verbosity=0
                             )
                    lgb.fit(X_train.iloc[train,    :],    Y_train.iloc[train],
eval_metric='logloss',eval_set= [(X_train.iloc[test, :],Y_train.iloc[test])])
        lgb_pred = lgb.predict(X_train.iloc[test, :])
        lgb_CM = confusion_matrix(lgb_pred, Y_train.iloc[test])
        lgb_AUC = roc_auc_score(Y_train.iloc[test], lgb_pred)
        result_auc.append(lgb_AUC)

    lgb_pred_test = lgb.predict(X_test)
    lgb_pred_test_prob = lgb.predict_proba(X_test)[:, 1]

    result = get_clf_eval(Y_test, lgb_pred_test, lgb_pred_test_prob, lgb, df,
folder_path)
    get_feature_importance(X_train, lgb, folder_path)
    get_confusion_matrix(Y_test, lgb_pred_test, "LGB", folder_path)

    return lgb,lgb_pred_test_prob, result



def          custom_train(X_train,Y_train,X_test,Y_test,folder_path,model_list,
optuna_trial_list, perfomances_list):
    perfomances_list = perfomances_list.insert(0, 'Model')
    df = pd.DataFrame(columns=perfomances_list)
    for model_name,optuna_trial in zip(model_list,optuna_trial_list):
        if "LR" in model_name:
            study1 = optuna.create_study(direction='minimize')
            study1.optimize(lambda trial: objective_lr(trial, X_train, Y_train,
X_test, Y_test), n_trials=optuna_trial,show_progress_bar=True)
            LR_parms = study1.best_params
            LR_best_trial = study1.best_trial
               lr,lr_pred_test_prob,  result  =  best_study_LR(X_train,  X_test,
Y_train, Y_test, LR_parms, df, folder_path)
```

```
            lr_filename = os.path.join(folder_path,'lr.model')
            pickle.dump(lr, open(lr_filename, 'wb'))

                              explainer    =    shap.Explainer(lr,    X_train,
feature_names=X_train.columns)
            shap_values = explainer(X_test)

            shap.summary_plot(shap_values, X_test, max_display=51, show=False)
            plt.tight_layout()
                              plt.savefig(folder_path+'/SHAP_lr_beeswarm.png',
dpi=300,pad_inches=0.4)
            plt.close()

            shap.plots.bar(shap_values, max_display=51, show=False)
            plt.tight_layout()
                         plt.savefig(folder_path   +   '/SHAP_lr_values.png',
dpi=300,pad_inches=0.4)
            plt.close()

        elif "SVM" in model_name:
            study2 = optuna.create_study(direction='minimize')
            study2.optimize(lambda trial: objective_svm(trial, X_train, Y_train,
X_test, Y_test), n_trials=optuna_trial,show_progress_bar=True)
            SVC_parms = study2.best_params
             svc,svc_pred_test_prob, result = best_study_SVM(X_train, X_test,
Y_train, Y_test, SVC_parms, result, folder_path)

            svc_filename = os.path.join(folder_path,'svm.model')
            pickle.dump(svc, open(svc_filename, 'wb'))

                              explainer    =    shap.Explainer(svc,    X_train,
feature_names=X_train.columns)
            shap_values = explainer(X_test)

            shap.summary_plot(shap_values, X_test, max_display=51, show=False)
            plt.tight_layout()
                       plt.savefig(folder_path   +   '/SHAP_SVC_beeswarm.png',
dpi=300,pad_inches=0.4)
            plt.close()
            shap.plots.bar(shap_values, max_display=51, show=False)
            plt.tight_layout()
                         plt.savefig(folder_path   +   '/SHAP_SVC_values.png',
dpi=300,pad_inches=0.4)
            plt.close()


        elif "RF" in model_name:
```

```
            study3 = optuna.create_study(direction='minimize')
            study3.optimize(lambda trial: objective_rf(trial, X_train, Y_train,
X_test, Y_test), n_trials=optuna_trial,show_progress_bar=True)
            rf_parms = study3.best_params
               rf,rf_pred_test_prob,  result  =  best_study_RF(X_train,  X_test,
Y_train, Y_test, rf_parms, result, folder_path)

            rf_filename = os.path.join(folder_path,'rf.model')
            pickle.dump(rf, open(rf_filename, 'wb'))

                              explainer     =     shap.TreeExplainer(rf,X_test,
feature_names=X_train.columns)
            shap_values = explainer.shap_values(X_test, check_additivity=False)
            shap_values_bar = explainer(X_test, check_additivity=False)

            parameters = {'ytick.labelsize': 1}
            plt.rcParams.update(parameters)
            print(shap_values.shape)
             shap.summary_plot(shap_values_bar[:,:,1], X_test, max_display=51,
show=False)
            plt.tight_layout()
                        plt.savefig(folder_path    +    '/SHAP_RF_beeswarm.png',
dpi=300,pad_inches=0.4)
            plt.close()

            shap.plots.bar(shap_values_bar[:,:,1],max_display=51, show=False)
            plt.tight_layout()
                         plt.savefig(folder_path    +    '/SHAP_RF_values.png',
dpi=300,pad_inches=0.4)
            plt.close()

        elif "XGB" in model_name:
            study4 = optuna.create_study(direction='minimize')
            study4.optimize(lambda trial: objective_xgb(trial, X_train, Y_train,
X_test, Y_test), n_trials=optuna_trial,show_progress_bar=True)
            XGB_parms = study4.best_params
             xgb,xgb_pred_test_prob, result = best_study_XGB(X_train, X_test,
Y_train, Y_test, XGB_parms, result, folder_path)

            xgb_filename = os.path.join(folder_path,'xgb.model')
            pickle.dump(xgb, open(xgb_filename, 'wb'))

            explainer = shap.TreeExplainer(xgb)
            shap_values = explainer(X_test, check_additivity=False)

            shap.summary_plot(shap_values, X_test, max_display=51, show=False)
            plt.tight_layout()
                       plt.savefig(folder_path    +    '/SHAP_XGB_beeswarm.png',
```

```
dpi=300,pad_inches=0.4)
            plt.close()

            shap.plots.bar(shap_values, max_display=51, show=False)
            plt.tight_layout()
                         plt.savefig(folder_path    +    '/SHAP_XGB_values.png',
dpi=300,pad_inches=0.4)
            plt.close()

        elif "LGB" in model_name:
            study5 = optuna.create_study(direction='minimize')
            study5.optimize(lambda trial: objective_lgb(trial, X_train, Y_train,
X_test, Y_test), n_trials=optuna_trial,show_progress_bar=True)
            lgb_parms = study5.best_params
             lgb,lgb_pred_test_prob, result = best_study_lgb(X_train, X_test,
Y_train, Y_test, lgb_parms, result, folder_path)

            lgb_filename = os.path.join(folder_path,'lgb.model')
            pickle.dump(lgb, open(lgb_filename, 'wb'))

                                explainer     =     shap.Explainer(lgb,X_train,
feature_names=X_train.columns)
            shap_values = explainer(X_test, check_additivity=False)

            parameters = {'ytick.labelsize': 1}
            plt.rcParams.update(parameters)

            shap.summary_plot(shap_values, X_test, max_display=51, show=False)
            plt.tight_layout()
                       plt.savefig(folder_path    +    '/SHAP_LGB_beeswarm.png',
dpi=300,pad_inches=0.4)
            plt.close()

            shap.plots.bar(shap_values, max_display=51, show=False)
            plt.tight_layout()
                         plt.savefig(folder_path    +    '/SHAP_LGB_values.png',
dpi=300,pad_inches=0.4)
            plt.close()

        else:
            print("This model is not available!!")

    result.to_excel(os.path.join(folder_path,'results.xlsx'), index=False)
```

```python
def main():
    scaler_df = custom_scaler(variables, scaler)
      X_train,  X_test,  Y_train,  Y_test  =  custom_target(scaler_df,  target,
target_name, valid_size)
    class_ratio(Y_train, Y_test)

    custom_train(X_train,Y_train,X_test,Y_test,folder_path,model_list,
optuna_trial_list, perfomances_list)


if __name__ == "__main__":
    main()
```
